# Developing an adapted Charlson comorbidity index for ischemic stroke outcome studies

**DOI:** 10.1186/s12913-019-4720-y

**Published:** 2019-12-03

**Authors:** Ruth E. Hall, Joan Porter, Hude Quan, Mathew J. Reeves

**Affiliations:** 1 ICES, 2075 Bayview Ave., G-Wing, Toronto, Ontario M4N 3M5 Canada; 2 Institute for Health Policy Management and Evaluation, Dalla Lana School of Public Health, University of Toronto, Ontario, Toronto, Canada; 3 Department of Community Health Sciences, Faculty of Medicine, University of Calgary, Alberta, Calgary, Canada; 4 Department of Epidemiology, Michigan State University, East Lansing, MI USA

**Keywords:** Charlson comorbidity, Ischemic stroke, Administrative data, Risk adjustment, Mortality

## Abstract

**Background:**

The Charlson comorbidity index (CCI) is commonly used to adjust for patient casemix. We reevaluated the CCI in an ischemic stroke (IS) cohort to determine whether the original seventeen comorbidities and their weights are relevant.

**Methods:**

We identified an IS cohort (*N* = 6988) from the Ontario Stroke Registry (OSR) who were discharged from acute hospitals (*N* = 100) between April 1, 2012 and March 31, 2013. We used hospital discharge ICD-10-CA data to identify Charlson comorbidities. We developed a multivariable Cox model to predict one-year mortality retaining statistically significant (*P* < 0.05) comorbidities with hazard ratios ≥1.2. Hazard ratios were used to generate revised weights (1–6) for the comorbid conditions. The performance of the IS adapted Charlson comorbidity index (ISCCI) mortality model was compared to the original CCI using the c-statistic and continuous Net Reclassification Index (cNRI).

**Results:**

Ten of the 17 Charlson comorbid conditions were retained in the ISCCI model and 7 had reassigned weights when compared to the original CCI model . The ISCCI model showed a small but significant increase in the c-statistic compared to the CCI for 30-day mortality (c-statistic 0.746 vs. 0.732, *p* = 0.009), but no significant increase in c-statistic for in-hospital or one-year mortality. There was also no improvement in the cNRI when the ISCCI model was compared to the CCI.

**Conclusions:**

The ISCCI model had similar performance to the original CCI model. The key advantage of the ISCCI model is it includes seven fewer comorbidities and therefore easier to implement in situations where coded data is unavailable.

## Background

The Charlson comorbidity index (CCI) is the most commonly used casemix adjustment method in health outcome studies that use administrative data [1–13]. In short, using a population of general medical inpatients at one hospital over 30 years ago Charlson identified 17 comorbidites that were associated with one-year mortality and assigned weights to these conditions that when summed created an index that predicted mortality [11]. However, recent work by Quan et al. suggests reweighting of the original Charlson score may be appropriate as new data sources become available, and as the management and outcomes of patients with chronic conditions evolve [3]. In the case of stroke, although it remains a common reason for hospitalization, there have been significant declines in stroke hospitalization and mortality rates over the past fifteen years [13–18]. Considering the median age of stroke patients is 75 years, many of these patients have a high pre-existing comorbid burden that can impact mortality making adjustment for differences in comorbidity burden critical when comparing quality of care across provider groups, regions, and countries [13, 17]. The validity of the CCI to adjust for comorbidity in stroke care, was done over 10 years ago, using ICD-9-CM coded inpatient adminstrative data from acute stroke patients across nine Vetern Affairs hospitals [4]. These circumstances support testing the accuracy of the original Charlson weights in a contemporary cohort of ischemic stroke inpatients.

We sought to determine whether the original Charlson comorbidities and corresponding weights were applicable in a cohort of ischemic stroke patients in Ontario by applying Quan et al’s approach to re-calculating Charlson comorbidity weights [3]. We also examined the impact of including atrial fibrillation into the CCI given it is a highly prevalent comorbid condition for ischemic stroke patients and is associated with higher mortality [19–23].

## Methods

### Setting

This is a retrospective population-based cohort of adults hospitalized with acute ischemic stroke in Ontario, Canada (population 13.3 million). Ontario has a publicly funded health care system that provides coverage of all medically necessary services delivered in hospitals, emergency departments and physician offices and prescription medications for adults aged 65 years and older.

### Data sources

The Ontario Stroke Registry’s (OSR) provincial acute stroke audit was used to identify hospitalized ischemic stroke cases. The stroke audit database consists of audited medical records of a population-based sample of patients aged 18 years and older discharged from provincial hospitals between April 1, 2012 and March 31, 2013 with transient ischemic attack (TIA), ischemic stroke, or intracerebral hemorrhage. Details of the audit methodology have been reported previously [18]. The audit was conducted by trained research personnel with access to stroke specialists for consultation and validation by duplicate chart abstraction has demonstrated excellent agreement for stroke type and stroke severity [24]. We restricted the cohort to patients with ischemic stroke. However because several of the 17 Charlson comorbidity conditions that make up the CCI were not captured in the chart audit, we linked the OSR ischemic cohort to the Discharge Abstract database (DAD) and Same-day Surgery database (SDS) using unique encoded identifiers to identify all Charlson comorbidities as well as atrial fibrillation. We included atrial fibrillation as a comorbid condition given its association with stroke mortality [19, 20, 22].

The DAD and SDS is compiled and maintained by the Canadian Institute for Health Information (CIHI) and contains information on all inpatient and day-surgery discharges from acute care hospitals in the province. Data elements in the DAD and SDS include the most responsible diagnosis and up to 24 other diagnoses that are coded according to the International Classification of Diseases, 10th revision, Canada (ICD-10-CA) standard. For each ischemic stroke record we determined the presence or absence of individual Charlson comorbidities as defined by Quan et al ICD-10-CA codes and the atrial fibrillation ICD-10-CA code (see Additional file 1) found in the DAD and SDS databses using a two-year look-back of DAD and SDS records and for index event (i.e., hosptialization for acute stroke), all the diagnoses coded and any diagnosis type (most responsible, pre-admission or post-admission) were also included in the identification process with exception of cerebrovascular disease [25–27]. In-hospital death was captured in the DAD and to obtain one-year and 30-day mortality we linked the ischemic stroke cohort to the Ontario Registered Persons Database (RPDB), a database of health insurance plan registrants that includes date of death. All linkages were done using unique encoded identifiers and analyzed at ICES.

### Analysis

The ischemic stroke cohort was randomly split into two cohorts; a test cohort (2/3rd) and validation cohort (1/3rd). Descriptive analysis compared characteristics of test and validation cohorts. In the test cohort we developed a multivariable Cox-proportional hazards model (Cox-PH) with one-year mortality as the dependent variable; predictor variables included age groups (< 45 years, 45–54, 55–64, 65–74, 75–84, 85+), sex, individual Charlson comorbidities as well as atrial fibrillation. Charlson comorbidities and atrial fibrillation were considered if they had a frequency of at least 10 patients and a bivariate association with one-year mortality of *p* < = 0.15. After adding all eligible comorbidites to the model we retained conditions with hazard ratios (HR) greater or equal to 1.2 and *p*-value < 0.05. The revised comorbidity weights were assigned to the individual comorbidities according to the following algorithm as developed by Quan: a weight of 1 for risk-adjusted hazard ratio of > = 1.2 but < 1.5, a weight of 2 for a hazard ratio of > = 1.5 but < 2.5, a weight of 3 for a hazard ratio of > = 2.5 but < 3.5, a weight of 4 for a hazard ratio of > = 3.5 but < 4.5, a weight of 5 for a hazard ratio of > = 4.5 but < 6 and a weight of 6 for a hazard ratio > = 6 [3]. These steps were repeated with the addition of atrial fibrillation. In summary, we 1) re-weighted the CCI from an ischemic stroke cohort (ISCCI); and 2) re-weighted the CCI adding atrial fibrillation as a comorbid condition (ISCCI-AF). In the validation cohort, we 1) assigned the original Charlson weights to the 17 comorbidities in the ischemic stroke cohort; 2) assigned the ISCCI recalibrated weights; and 3) assigned the ISCCI-AF weights. We then used logistic regression to model three outcomes; in-patient, 30 day and 1 year mortality following ischemic stroke adjusting for age, sex and each of the three comorbidity indices: the original CCI, the ischemic stroke Charlson index (ISCCI) and the ISCCI-AF; which created a total of nine separate models. We used the Hosmer-Lemeshow goodness-of-fit test to assess model calibration and, the c-statistic to assess model discrimination. A c-statistic between 0.7–0.8 indicates reasonable model and > 0.8 is considered a strong model [28]. We also computed the continuous net reclassification index (cNRI) to determine whether the ISCCI model had an improved ability to predict risk of death compared to the original Charlson index (ISCCI vs CCI). The cNRI measures the improvement in correctly classifying patients as high (or low) risk for death by calculating the sum of the differences in the estimated probability of net upward reclassification of death and the estimated probability of net downward reclassification for no death [29]. We used SAS Enterprise Guide, version 6.1 for all analyses (SAS Institute, Cary, NC).

## Results

Characteristics of ischemic stroke patients in the test and validation cohorts were similar (Table 1). The majority of ischemic stroke patients had mild stroke (59.7% in the test cohort and 58.6% in the validation). In addition to the most responsible diagnosis of ischemic stroke, a median of four diagnosis codes were found in the index hospitalization record in both cohorts. All-cause death within one year of admission was similar for the test (24.6%) and validation (23.4%) cohorts. The median survival time among those who died within 1 year was 30 days and 32 days, in the 2 cohorts, respectively. The frequency of individual comorbid conditions was similar with the exception of mild liver disease where the validation cohort had a slightly higher proportion (Table 1). The most frequently reported comorbidities were diabetes with complication (28%), atrial fibrillation (27%), and hemi or paraplegia (17%).

**Table 1:** Characteristics of Ischemic Stroke test and validation cohorts, Ontario Stroke Audit, 2012/13

Characteristic	Test	Validation
	*n* = 4657	n = 2331
Sex (%)		
Male	2362 (50.7)	1201 (51.5)
Female	2295 (49.3)	1130 (48.5)
Age Group (years) (%)		
18–64	1166(25.0)	588 (25.2)
65–74	983 (21.1)	517 (22.2)
75–84	1443 (31.0)	646 (27.7)
85+	1065 (22.9)	580 (24.9)
CNS category (%)		
Mild	2778 (59.7)	1367 (58.6)
Moderate	939 (20.2)	485 (20.8)
Severe	727 (15.6)	369 (15.8)
Missing	213 (4.6)	110 (4.7)
Mean (median) # diagnoses fields completed	4.5 (4)	4.4 (4)
Inpatient death (%)	480 (10.3)	220 (9.4)
Death <= 30 days (%)	579 (12.4)	265 (11.4)
Death < 366 days (%)	1145 (24.6)	545 (23.4)
Median survival (days) (IQR)	30 (8–125)	32 (8–138)
Frequency of Charlson comorbid condition (%)		
Myocardial infarction	331 (7.1)	177 (7.6)
Congestive heart failure	475 (10.2)	219 (9.4)
Peripheral vascular disease	160 (3.4)	91 (3.9)
Cerebrovascular disease	260 (5.6)	137 (5.9)
Dementia	357 (7.7)	182 (7.8)
Chronic obstructive pulmonary disease	336 (7.2)	146 (6.3)
Rheumatic disease	50 (1.1)	27 (1.2)
Peptic ulcer disease	58 (1.3)	29 (1.2)
Mild liver disease	21 (0.5)	20 (0.9)
Diabetes without complications	107 (2.3)	50 (2.2)
Diabetes with complications	1347 (28.9)	717 (30.8)
Hemi or paraplegia	763 (16.4)	375 (16.1)
Renal disease	260 (5.6)	106 (4.6)
Primary cancer	175 (3.8)	99 (4.3)
Moderate or severe liver disease	9 (0.2)	7 (0.3)
Metastatic cancer	77 (1.7)	39 (1.7)
HIV/AIDS	< 6 (< 0.2)	< 6 (< 0.3)
Atrial fibrillation (not a Charlson comorbidity)	1262 (27.1)	627 (26.9)
Mean (s.d.) CCI– 17 comorbidities	1.7 (1.8)	1.7 (1.8)
Mean (s.d.) ISCCI– 10 comorbidities	NA	1.3 (1.6)

Sources: Ontario Stroke Audit 2012/13, Registered Persons Database, Discharge Abstract Database

CNS – Canadian Neurological Scale, a measure of stroke severity and categorized as mild (> = 8), moderate [5–7], or severe (<=4) [30]

HIV/AIDS – human immunodeficiency virus/acquired immunodeficiency syndrome

CCI – Charlson Comorbidity Index

ISCCI – Ischemic Stroke Comorbidity Index

s.d. – standard deviation

NA – not applicable; index was developed on the test cohort

**Fig. 1:**
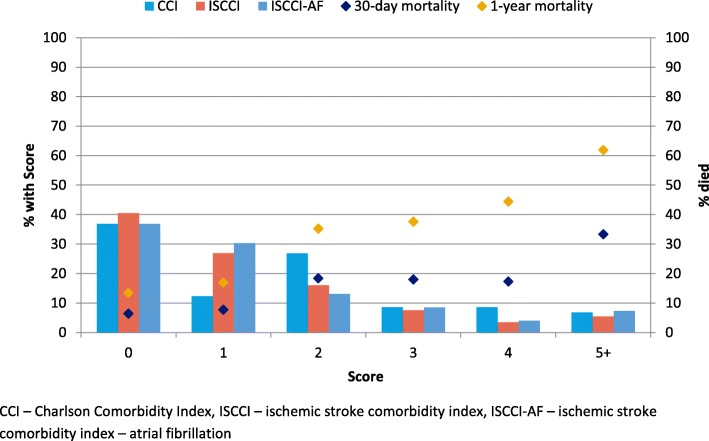
Distribution of comorbidity score, by index, and mortality associated with ISCCI among the validation cohort (*N* = 2331), April 1, 2012 to March 31, 2013

Comorbidity hazard ratios and weights for ISCCI derived from the test cohort are shown in Table 2. For comparison purposes, the original Charlson weights are also shown [11]. There was no difference in weights when atrial fibrillation (ISCCI-AF) was included in the model compared to the ISCCI model weights except cerebrovascular disease became non-significant and atrial fibrillation became significant with a weight of 1 (data not shown). Ten of the 17 Charlson comorbid conditions in the ISCCI were statistically significant (i.e, a HR ≥ 1.2). Compared to Charlson weights, the ISCCI weights were one point higher for four conditions (myocardial infarction (MI), congestive heart failure (CHF), dementia, rheumatologic disease), remained the same for three conditions (cerebrovascular disease (CVD), chronic obstructive pulmonary disease (COPD), any malignancy,) and, one point lower for three conditions (diabetes with chronic complications, hemi or paraplegia, metastatic solid tumor). Figure 1 compares the frequency of CCI scores with ISCCI, and ISCCI-AF scores in the validation cohort. The distribution of scores shows a similar proportion of patients with 0 (~ 38%) and 5 and higher scores (~ 6%), but the two new models, had more than double the proportion of patients with a score of 1 (26.9, 30.3%, vs 12.3%), and a smaller proportion of patients with scores of 2 and 4 compared to the original CCI. Figure 1 also illustrates the observed 30-day and one-year mortality. As the comorbidity score increases, the proportion of 1 year mortality correspondingly increases. For example, among patients with an ISCCI of 5 or greater 33.3% died within 30 days and 61.9% within one year.

**Table 2:** Comorbidity weights derived from the ischemic stroke test cohort (April 1, 2012 to March 31, 2013) compared with weights of the original Charlson comorbidity index [11]

Ischemic stroke weight (ISCCI)	Comorbid condition	Charlson Weight
2	Myocardial infarction	1
2	Congestive heart failure	1
0	Peripheral vascular disease	1
1	Cerebrovascular disease	1
2	Dementia	1
1	Chronic obstructive pulmonary disease	1
2	Rheumatologic disease	1
–	Peptic ulcer disease	1
–	Mild liver disease	1
–	Diabetes without chronic complications	1
1	Diabetes with chronic complications	2
1	Hemiplegia or paraplegia	2
0	Renal disease	2
2	Primary Cancer (any malignancy)	2
–	Moderate or severe liver disease	3
5	Metastatic solid tumor	6
–	AIDS/HIV	6
19	Maximum comorbidity score	29

- Not included in the Cox proportional hazard model as did not achieve threshold in bivariate analysis

Calculation of the maximum score is based on a hierarchy where diabetes with complications supersedes diabetes without complications; moderate or severe liver disease supersedes mild liver disease; and metastatic solid tumor supersedes any malignancy

Table 3 shows the calibration and predictive accuracy of modelling the probability of death in-hospital, within 30 days or 1 year of admission for the three models. For all mortality outcomes, thec-statistics for the ISCCI models were higher compared to the CCI model. For example, for 30-day mortality the c-statistic for the original CCI was 0.732 compared to 0.746, for the ISCCI *p* = 0.009. However the difference between the CCI and the ISCCI model were negligible and non-significiant for in-hospital death and one-year (0.722 vs 0.729 *p* = 0.343 and 0.760 vs 0.764, *p* = 0.398, respectively). Including atrial fibrillation (ISCCI-AF) did not improve the ISCCI model discrimination. The cNRI analysis did not include the ISCCI-AF model given the lower c-statistic compared to the ISCCI. The cNRI showed the ISCCI model compared to the CCI model did not improve patient net mortality risk reclassification (Table 3).

**Table 3:** Comparison of Model performance among ischemic stroke patients in the validation cohort (*N* = 2331, discharged between April 1, 2012 and March 31, 2013)

Outcome	Model	H-L X^2†^ (df)	*P* value	C-statistic (95% CI)	*P* value*	cNRI^¥^ (95% CI)	*P* value*
In-hospital mortality	Age/sex adjusted CCI	9.56 (8)	0.297	0.722 (0.688, 0.756)			
	Age/sex adjusted ISCCI	8.35 (8)	0.400	0.729 (0.695, 0.762)	0.343	−0.121 (−0.260,0.017)	0.08
	Age/sex adjusted ISCCI-AF	22.01 (8)	0.005	0.720 (0.686, 0.753)	–	–	
30-day mortality	Age/sex adjusted CCI	10.42 (8)	0.236	0.732 (0.700, 0.763)			
	Age/sex adjusted ISCCI	11.65 (8)	0.234	0.746 (0.715, 0.777)	0.009	0.041 (−0.087,0.170)	0.53
	Age/sex adjusted ISCCI-AF	12.95 (8)	0.113	0.737 (0.706, 0.768)		–	
One-year mortality	Age/sex adjusted CCI	7.38 (8)	0.391	0.760 (0.738, 0.783)			
	Age/sex adjusted ISCCI	7.33 (8)	0.501	0.764 (0.741, 0.786)	0.398	−0.086 (−0.181,0.010)	0.08
	Age/sex adjusted ISCCI-AF	15.63 (8)	0.075	0.755 (0.732, 0.777)		–	

*H-L* Hosmer-Lemeshow, *cNRI* continuous net reclassification improvement, *CCI* Charlson comorbidity index, *ISCCI* Ischemic stroke Charlson comorbidity index, *ISCCI-AF* Ischemic stroke Charlson comorbidity index with atrial fibrillation included in the development model

†chi-square

*p-value for Area Under the Curve (AUC) and cNRI difference between age&sex adjusted CCI and age&sex adjusted ISCCI

¥difference between age/sex adjusted CCI and age/sex adjusted ISCCI

## Discussion

Using a large population-based ischemic stroke cohort discharged from acute hospitals in Ontario, Canada between April 1, 2012 and March 31, 2013, we examined the association between the Charlson comorbidities and mortality to determine which Charlson comorbidities are relevant among ischemic stroke patients. Similar to other studies, we found several Charlson comorbidities were not signifinicant predictors of one-year mortality in ischemic stroke patients [3, 6, 31–33]. In fact, we found just ten of the 17 Charlson comorbidities to be associated with one-year mortality following an acute ischemic stroke. The very low prevalence of moderate or severe liver disease and AIDS/HIV (*n* < 10) meant they were not considered for the ISCCI. Of the remaining Charlson comorbidities, peripheral vascular disease, peptic ulcer disease, mild liver disease, diabetes without complications, renal disease, were not associated with one-year mortality in our ischemic stroke cohort (*p* > 0.05).

Although the ISCCI model demonstrated marginal improved model performance (< 2% increase in c-statistic) compared with the original CCI, the extent of model improvement is similar to other studies comparing performance of revised CCI models to the originial CCI [3, 7, 31, 32]. Not surprisingly, there was little difference in model performance between the ISCCI and ISCCI-AF models across all outcomes given no difference in the total number of comorbidities and the corresponding weights. Additionally, the ISCCI didn’t result in significant gain or loss in net patient mortality risk reclassifications.

A criticism of the CCI derived from administrative databases is the concern over misclassifying complications as comorbidities [5, 26, 34]. Although the CIHI DAD and SDS databases have diagnosis type fields to allow differentiation between a pre-admission condition and conditions that developed during the hospitalization, reabstraction studies have found modest validity for the diagnosis type field and therefore distinguishing conditions that arise as a consequence of natural disease progression from complications of care is limited [27, 34]. However, most Charlson comorbidities, with the possible exception of hemi- or paraplegia, would not be considered complications in ischemic stroke patients. The prevalence of existing hemi- or paraplegia among acute stroke patients in the OSR is ~ 2% (data not shown). We examined the 1138 IS patients identified as having hemi- or paraplegia and determined all were coded in the index hospitalization record. The majority (87.7%) classified hemi- or paraplegia as a pre-existing condition, 2.8% a post admission condition or complication and 9.5% a secondary diagnosis. A secondary diagnosis does not meet the requirement for determining comorbidity, ie., capturing a symptom of the stroke [27]. CIHI DAD data quality studies have revealed overall coder agreement of diagnosis type was 76%, ranging from 65% for post-admission diagnoses, 67% for pre-existing diagnoses to 86% for most responsible diagnosis [27]. Given coders are limited to physician documentation only when assigning diagnosis types and, the way physicians document is to record conditions relevant to patient treatment/management, it is not surprising coder assignment of diagnosis type is challenging [35, 36].

We suspect the coding of hemi- or paraplegia may be caputuring either; 1) acute stroke associated symptoms or, 2) prior stroke given the low prevalence of prior stroke in our cohort (cerebrovascular disease ~ 6%). If, hemi- paraplegia coding in the index acute stroke event is capturing the symptoms of the acute stroke this may reflect stroke severity and given stroke severity is not available in administrative databases, it may be reasonable to include hemi- and paraplegia in an administrative database derived risk-adjustment model.

Stroke severity is strongly associated with mortality and recommended to be included in risk adjustment models [37]. Given, our ischemic stroke cohort was from the OSR and the OSR captures stroke severity through the Canadian Neurological Scale, we added stroke severity to the ISCCI model as an additional covariate and model performance improved substantially (c = 0.855 vs 0.746, for 30-day mortality, data not shown).

If we consider the 87.7% of the 1138 IS patients with hemi- or paraplegia coded as a pre-existing condition to be a result of a previous stroke; and combine it with the cerebrovascular disease prevalence, (*n* = 397) the prevalence of previous stroke would be ~ 20% and is within the range reported in the literature [17, 18, 38–40]. Further investigation of including hemi- or paraplegia diagnostic codes into algorithms to identify prior stroke in ICD coded databases is warranted.

Our study is not without limitations, firstly, we only examined mortality and our findings may not apply to other outcomes including length of stay, cost, readmission and patient functional outcomes. We also limited comorbidity identification to acute inpatient and same-day surgery hospital-based claims with a two-year lookback. However, we found little gain in model performance when we used a three-year lookback (data not shown) and a longer lookback was not examined . Secondly, the accuracy of ICD codes and number of diagnostic code fields available and or completed to capture comorbidity conditions is jurisdictional dependent [3, 41]. In Ontario, the low prevalence of comorbidities in administrative databases compared with clinical data obtained in reabstraction studies has been reported, although when a lookback period was applied, prevalence improved for several comorbidities [42–46]. Other stroke-related comorbidities associated with mortality and higher population attributable risk of stroke, such as obesity, smoking and hypertension were not examined given the low prevalence and unreliable coding of smoking and obesity in hospital-based administrative data and hypertension has been shown to be negatively associated with mortality [23, 47–49]. Additionally, we did not examine other administrative data sources like physician billing, emergency department, drug and laboratory databases for the comorbidity history of our cohort. However, little gain in model performance has been observed when physician billing data were included with hospitalization data [5, 50]. We did not use the OSR to identify comorbidities due to the inability to map one to one the 17 Charlson comorbidities (e.g., the OSR does not distinguish between diabetes with and without complications, mild vs moderate/severe liver disease and does not capture history of rheumatic disease) and our intended focus was on administrative data derived CCI not comparing a claims-based CCI to a medical record-based CCI. The ability to identify comorbidities in various databases such as-physician claims, laboratory, diagnostic imaging and drug databases and electronic medical records, is worthy of future research especially with the growing access to these data sources and increasing computational capacity and advanced analytical techniques like machine learning algorirthms that allow for the integration of time-dependent data. Finally, our findings are based on Ontario administrative data within the context of a universal health care system with mandatory hospitalization data submission and processes for error checking; therefore our results may not be generalizable to other settings or populations. Despite this, our findings are from a large, province-wide sample of ischemic stroke in-patients with complete follow-up for deaths of varying time frames.

## Conclusion

We have shown the ISCCI model had similar performance to the original CCI model and therefore in the context of an ischemic stroke cohort the CCI remains a valid measure of comorbidity when using administrative data. The key advantage of the ISCCI model is it includes seven fewer comorbidities (10 vs 17) and therefore easier to implement in situations where coded data is unavailable (e.g. chart reviews, clinical trials, surveys and clinical registries).

## Supplementary information

**Additional file 1.** ICD-10-CA codes for comorbidity conditions.

## Data Availability

The full data set creation plan is available from the corresponding author upon request. The datasets from this study are held securely in coded form at ICES. While data sharing agreements prohibit ICES from making the datasets publicly available, access may be granted to those who meet pre-specified criteria for confidential access, available at www.ices.on.ca/DAS.

## Abbreviations

CCI: Charlson comorbidity index
CHF: Congestive heart failure
CIHI: Canadian Institute for Health Information
cNRI: continuous Net Reclassification Index
COPD: Chronic obstructive pulmonary disease
CVD: Cerebrovascular disease
DAD: Discharge abstract database
ICD: International classification of diseases
ICD-10-CA: International classification of diseases, 10th revision, Canada
ISCCI: Ischemic stroke comorbidity index
ISCCI-AF: Ischemic stroke comorbidity index-atrial fibrillation
MI: Myocardial infarction
OSR: Ontario stroke registry
RPDB: Registered Persons Database (RPDB)
SDS: Same day surgery database
TIA: Transient ischemic attack
