# Supplementary material for: Developing an adapted Charlson comorbidity index for ischemic stroke outcome studies
Source: BMC Health Serv Res. 2019 Dec 3;19:930. doi: 10.1186/s12913-019-4720-y (PMC6892203; doi:10.1186/s12913-019-4720-y)
Supplement: Supplementary file 1 — Additional file 1. ICD-10-CA codes for comorbidity conditions. [file 12913_2019_4720_MOESM1_ESM.docx]

**Additional file 1**

**ICD-10-CA codes for comorbidity conditions**

| **Comorbidity condition** | **ICD-10-CA code** |
| --- | --- |
| Acute Myocardial Infarction | I21, I22, I252 |
| Congestive Heart Failure | I099, I255, I420, I425-I429, I43, I50, P290 |
| Peripheral Vascular Disease | I70, I71, I731, I738, I739, I771, I790, I792, K551, K558, K559, Z958, Z959 |
| Cerebrovascular Disease | G45, G46, H340, I60-I69 |
| Dementia | F00-F03, F051, G30, G311 |
| Chronic Obstructive Pulmonary Disease or other Respiratory diseases | I278, I279, J40-J47, J60-J67, J684, J701, J703 |
| Rheumatic-like Diseases | M05, M06, M315, M32-M34, M351, M353, M360 |
| Ulcers of the Digestive System | K25-K28 |
| Liver Disease - Mild | B18, K700-K703, K709, K713-K715, K717, K73, K74, K760, K762-K764, K768, K769, Z944 |
| Diabetes - No Chronic Complications | E100, E101, E106, E108, E109, E110, E111, E116, E118, E119, E120, E121, E126, E128, E129, E130, E131, E136, E138, E139, E140, E141, E146, E148, E149 |
| Diabetes with Chronic Complications | E102-E105, E107, E112-E115, E117, E122-E125, E127, E132-E135, E137, E142-E145, E147 |
| Hemiplegia or Paraplegia | G041, G114, G801, G802, G81, G82, G830-G834, G839 |
| Renal (Kidney) Disease | N032-N037, N052-N057, N18, N19, N250, Z490-Z492, Z940, Z992 |
| Cancer (No secondary found) | C00-C26, C30-C34, C37-C41, C43, C45-C58, C60-C76, C81-C85, C88, C90-C97 |
| Liver Disease - Moderate or Severe | I850, I859, I864, I982,K704, K711, K721, K729, K765, K766, K767 |
| Cancer (Metastatic -secondary) | C77-C80 |
| HIV / AIDS | B20-B22, B24 |
| Atrial fibrillation | I48 |

The following hierarchy exists in creating these indicator variables: diabetes with chronic complications trumps diabetes with no chronic complications; moderate or severe liver disease trumps mild liver disease; and metastatic cancer trumps cancer (no secondary found), i.e., a patient cannot simultaneously have mild and severe liver disease.
